# Weak association of anti-sperm antibodies and strong association of familial cryptorchidism/infertility with *HLA-DRB1*polymorphisms in prepubertal Ukrainian boys

**DOI:** 10.1186/1477-7827-9-129

**Published:** 2011-09-28

**Authors:** Maciej Kurpisz, Andriy Nakonechnyy, Wanda Niepieklo-Miniewska, Anna Havrylyuk, Marzena Kamieniczna, Beata Nowakowska, Valentyna Chopyak, Piotr Kusnierczyk

**Affiliations:** 1 Institute of Human Genetics Polish Academy of Sciences, Department of Reproductive Biology and Stem Cells, Strzeszynska 32, 60 479 Poznan, Poland; 2 Danylo Halytsky Lviv National Medical University, Department of Pediatric Surgery, Lviv, Ukraine; 3 Institute of Immunology and Experimental Therapy Polish Academy of Sciences, Department of Clinical Immunology, Laboratory of Immunogenetics and Tissue Immunology, Wroclaw, Poland; 4 Danylo Halytsky Lviv National Medical University, Department of Clinical Immunology and Allergology, Lviv, Ukraine; 5 Jan Długosz Pedagogical University, Faculty of Natural Sciences, Czestochowa, Poland

## Abstract

**Background:**

Cryptorchidism is a frequent syndrome occurring in 1-2% of males within the first year of age. Autoimmune reactions, particularly directed to testicular elements and/or spermatozoa have been found to be often associated with cryptorchidism. Therefore we investigated in this study the frequency of HLA class II alleles in order to recognize possible genetic predisposition for antisperm antibodies development in prepubertal boys with diagnosed cryptorchidism in Caucasoid population.

**Methods:**

Sixty prepubertal boys with cryptorchidism and sixty healthy boys were examined for anti-sperm antibodies by indirect immunobead test as well as for their *HLA-DRB1 *and -*DQB1 *alleles using DNA obtained from peripheral blood leukocytes. The typing of *HLA-DRB1 *and -*DQB1 *was performed by using PCR-SSP low resolution method.

**Results:**

Allele frequencies of *HLA-DRB1 *and *HLA-DQB1 *did not differ between boys with cryptorchidism and control boys. However, weakly significant differences in DRB1*04 (*p corrected *= 0.0475) and DQB1*06 (*p corrected *= 0.0385) were seen between cryptorchid patients with and without AsA, but none of these two patient groups differed significantly in *HLA *class II frequencies from controls except for AsA-negatives and HLA-DQB1*06 (*p corrected *= 0.0247). On the other hand, comparison of cryptorchid boys with familial cryptorchidism and/or infertility to control boys revealed highly significant (*p corrected *= 0.0006) difference in HLA-DRB*11 frequency, whereas boys with sporadic cryptorchidism did not differ from control. A much weaker, but still significant difference in DRB*11 frequency was also observed between boys with bilateral cryptorchidism and controls (*p corrected *= 0.037), whereas patients with unilateral cryptorchidism were not different from control in frequency of any *HLA-DRB1 *or -*DQB1 *allele tested.

**Conclusions:**

Predisposition to produce anti-sperm antibodies seems to be only weakly associated with *HLA *class II genes, although this question requires further study on much larger population sample. It is plausible that familial and sporadic cryptorchidism may present distinct genetic background. The same may, to lower extent, apply to bilateral and unilateral cryptorchidism.

## Background

Cryptorchidism is one of the most frequent pathologies in early childhood (1.4-8.4% in Caucasian term male births) in which one (unilateral) or both (bilateral) testes fail to descend into the ideal scrotal position. It is also a major risk for male infertility and for testicular malignancy in adulthood [1-3]. Genetic factors influencing susceptibility to cryptorchidism have not been clearly defined: despite the tendency for a familial aggregation of cryptorchidism, genetic abnormalities have been found only in a few patients [4], and several candidate genes have not been definitely confirmed [5,6].

Many sperm proteins do not arise until spermatogenesis starts at puberty. Nevertheless, anti-sperm antibodies (AsA) in serum samples from prepubertal boys were reported [7,8], and our earlier findings showed that cryptorchidism may play an important role in antibody formation at this age [9-11]. The human major histocompatibility complex Class II HLA molecules, by presenting antigens to helper T cells, play a decisive role in induction of antibody production [12]. Therefore, our aim was to examine whether there is any HLA class II-associated genetic predisposition for the development of AsA in prepubertal boys with cryptorchidism.

## Methods

### Patients

The study groups consisted of 60 prepubertal boys with cryptorchidism and 60 healthy boys, age and ethnicity-matched, from Western Ukraine. Control group under study was clinically found in good health condition and was no under vaccination regime when blood samples were drawn. The local Bioethical Committee of Medical Lviv University granted the permission for biological material to be drawn from the subjects for this experimental study. Participants provided a written consent for including their samples in the study. Characteristic of the patients is shown in Table 1. Clear recognition among the studied subgroups was made according to: sporadic (n = 42), familial (n = 17*; *one DNA sample was found not suitable for further studies due to poor DNA quality), unilateral (n = 40) and bilateral (n = 20) cryptorchidism. Male infertility was here defined as the lack of conception in natural procreation conditions for 2 years with maintained regular intercourse within a couple. Additionally, infertility has been recognized among the second to the third degree relationship while familial cryptorchidism was found among the first up to the third degree of relatives.

**Table 1:** Characteristics of patients with cryptorchidism.*

		AsA negative		AsA 1-10%		AsA>10%	
	**total**	**n**	**%**	**n**	**%**	**n**	**%**

<1 ano	3	3	100	0	0	0	0
1 a 4	29	17	58,6	7	24,1	5	17,2
5 a 9	16	9	56,3	3	18,8	4	25,0
10 a 14	12	8	66,7	3	25,0	1	8,3
Tanner							
P1	56	35	62,5	12	21,4	9	16,1
P2	3	1	33,3	1	33,3	1	33,3
P3	1	1	100,0	0	0,0	0	0,0
1-14 unilateral cryptorchidism				AsA (+)		13	65
1-14 bilateral cryptorchidism				AsA (+)		7	35
1-14 sporadic cryptorchidism				AsA (+)		17	27
1-14 familial cryptorchidism/infertility				AsA (+)		3	18
1-14 familial cryptorchidism				AsA (+)		3	30

*This cohort of individuals has been excluded from other possible interfering diseases of inflammatory/immunological nature; also from vaccination procedures and/or medical treatment.

### Anti-sperm antibody detection - IDIBT (indirect immunobead test)

Antisperm antibody detection in group with cryptorchidism has been performed prior to any surgical intervention and when determining the control population no positive results according to AsA presence by IDIBT assay were found.

The procedure has been previously described [10]. Briefly, motile sperm cells were isolated from semen of AsA-negative donors by swim-up technique. Sperm cells were washed in HAM F-10 medium supplemented with 1% of bovine serum albumin (BSA) and their concentration was adjusted to 25-30 × 10^6 ^per 1 mL. One hundred microliters of this suspension was mixed with 100 μl of a tested sample (serum) and 200 μl of HAM F-10/BSA, and incubated for 1 hr at 37°C. After incubation, the cells were washed in HAM F-10/BSA and resuspended to a concentration 25-30 × 10^6^/mL.

Five microliters of immunobead suspension (5 mg/ml) were mixed with 10 μl of final sperm suspension, covered with a coverslip and incubated in a moist chamber for 8 minutes at room temperature. The spermatozoa were observed in a phase-contrast microscope, at 400× magnification. Motile spermatozoa with at least 1 bead attached to their surface were considered as positive. Binding of immunobeads to the particular regions of a sperm cell (head, midpiece, tail) was also documented.

### Genomic DNA isolation

Peripheral blood samples were drawn on EDTA (ethylenediaminetetraacetic acid) and served for extraction of the genomic DNA. DNA was obtained by using a proteinase K digestion and commercially available DNA extraction kit (Isoquick Nucleic Extraction Kit, Invitek GmbH, Berlin, Germany). HLA class II alleles were typed using the polymerase chain reaction-sequence-specific primer (PCR-SSP) low resolution method. HLA-DRB1*, DQB1* typing was performed using the Dynal DRB1*, DQB1* typing kit (AllSet SSP *DRB1, DQB1*, Dynal Biotech.Ltd., Bromborough, UK) following manufacturer's instruction. PCR amplification was performed using genomic DNA (50 ng/μl), master mix (Dynal Biotech Ltd., Bromborough, UK), Platinum Taq polymerase (Invitrogen Brazil, 5 U/μl), and primers (24 sets for *HLA-DRB1 *and 8 for *HLA-DQB1*). The amplification profile consisted of denaturation 96°C (120 sec), next 10 cycles: 96°C (15 sec), 65°C (60 sec), and 20 cycles: 96°C (10 sec), 61°C (50 sec), 72°C (30 sec). After gel electrophoresis of PCR products in 2% agarose in 0.5 × TBE buffer (15 min at 10 V/cm), UV transilluminator (Vilber Lourmat, France) was used for visualization of the obtained amplification pattern.

### Statistical analysis

Statistical differences between prepubertal boys with cryptorchidism and healthy control subjects and among subgroups of cryptorchid boys were tested using chi-square analysis including Yates correction, and both tailed Fisher test as the numbers of patients after subdivision into different clinical categories were rather small. A level of *p_corrected _*< 0.05 was considered to be significant.

## Results

AsA-positive population of prepubertal boys here identified (Table 1) has been characterized according to unilateral (majority of cases) and bilateral cryptorchidism as well as sporadic and familial cases. Certain degree of surprise was noticed in respect to familial infertility where the number of positive for AsA case has been markedly lower (than expected) compared with a group of familial cryptorchidism. Due to the high specificity of detection test applied (IBT) there were no AsA positive individuals in control group under study.

Allele frequencies of *HLA-DRB1 *and *HLA-DQB1 *did not differ between boys with cryptorchidism and control boys (data not shown). However, weakly significant differences in DRB1*04 (*p_corrected _*= 0.0475, O.R. = 0.124) and DQB1*06 (*p_corrected _*= 0.0385, O.R. = 0.291) were seen between cryptorchid patients with and without AsA (Table 2), but none of these two patient groups differed significantly in *HLA *class II frequencies from controls (not shown) except for AsA-negative patients and HLA-DQB1*06 (*p_corrected _*= 0.0247, O.R. = 0.3229, C.I. = 0.1261-0.827).

**Table 2:** Comparison of HLA-DRB1 and -DQB1 allele frequencies in anti-sperm antibody (AsA)-positive and -negative patients with cryptorchidism.

Allele HLA-DRB1/DQB1	AsA negative N = 37		AsA positive N = 23				
	%	(N)	%	(N)	p/p_c _	OR	C.I
DRB1* 01	14	(10)	17	(7)			
DRB1* 15	7	(5)	15	(6)			
DRB1* 16	7	(5)	0	(0)			
DRB1* 03	10	(7)	10	(4)			
DRB1* 04	17	(12)	2,5	(1)	0.0221/0.0475	0.1239	0.01548- 0.9925
DRB1* 11	16	(11)	20	(8)			
DRB1* 12	1,5	(1)	5	(2)			
DR B1*13	10	(7)	10	(4)			
DR B1*14	3	(2)	2,5	(1)			
DRB1* 07	13	(9)	10	(4)			
DRB1* 08	1,5	(1)	2,5	(1)			
DRB1* 09	0	(0)	5	(2)			
DRB1* 10	0	(0)	0	(0)			
DQB1* 02	21	(15)	20	(8)			
DQB1* 03	43	(30)	32	(13)			
DQB1* 04	0	(0)	0	(0)			
DQB1* 05	27	(19)	22	(9)			
DQB1* 06	9	(6)	25	(10)	0.0187/0.0385	0.2813	0.09349-0.8461

*p_c _*= *p *corrected

On the other hand, comparison of cryptorchid boys with familial cryptorchidism and/or infertility to control boys revealed highly significant (*p_corrected _*= 0.0006, O.R. = 4.304) difference in HLA-DRB*11 frequency, whereas boys with sporadic cryptorchidism did not differ from control (Table 3). A much weaker, but still significant difference in DRB*11 frequency was also observed between boys with bilateral cryptorchidism and controls (*p_corrected _*= 0.037, O.R. = 3.000), whereas patients with unilateral cryptorchidism were not different from control in frequency of any *HLA-DRB1 *or -*DQB1 *allele tested (Table 4).

**Table 3:** Comparison of HLA-DRB1 and -DQB1 allele frequencies in patients with familial and sporadic cryptorchidism and in control individuals.

Allele HLA-DRB1/D	Controls N = 60		Cryptorchidism						
			Familial N = 17		Sporadic N = 42		p/p_c _	OR	**C.I**.
	%	(N)	%	(N)	%	(N)			
DRB1* 01	9	(11)	6	(2)	18	(15)			
DRB1* 15	10	(12)	9	(3)	12	(10)			
DRB1* 16	6	(7)	3	(1)	5	(4)			
DRB1* 03	8	(10)	15	(5)	11	(9)			
DRB1* 04	12	(14)	15	(5)	11	(9)			
DRB1* 11	10	(12)*	32	(11)*	12	(10)	*0.0012/0.0031	4.304	1.692-10.95
DRB1* 12	3	(4)	6	(2)	1	(1)			
DR B1*13	15	(18)	6	(2)	12	(10)			
DR B1*14	5	(6)	3	(1)	2	(2)			
DRB1* 07	12	(15)	6	(2)	12	(10)			
DRB1* 08	7	(9)	0	0	2	(2)			
DRB1* 09	0	(0)	0	0	2	(2)			
DRB1* 10	2	(2)	0	0	0	0			
DQB1* 02	18	(22)	18	(6)	21	(18)			
DQB1* 03	31	(37)	50	(17)	32	(27)			
DQB1* 04	5	(6)	0	0	0	0			
DQB1* 05	23	(28)	18	(6)	29	(24)			
DQB1* 06	22	(27)	15	(5)	18	(15)			


**Table 4:** Comparison of HLA-DRB1 and -DQB1 allele frequencies in patients with bilateral and unilateral cryptorchidism and in control individuals.

Allele HLA-DRB1/DQB1	Controls N = 60		Cryptorchidism						
			Bilateral N = 20		Unilateral N = 40		p/p_c _	OR	**C.I**.
	%	(N)	%	(N)	%	(N)			
DRB1* 01	9	(11)	15	(6)	15	(12)			
DRB1* 15	10	(12)	7,5	(3)	14	(11)			
DRB1* 16	6	(7)	2,5	(1)	5	(4)			
DRB1* 03	8	(10)	5	(2)	14	(11)			
DRB1* 04	12	(14)	10	(4)	11	(9)			
DRB1* 11	10	(12)*	25	(10)*	13	(10)	*0.0171/0.0369	3.000	1.181-7.617
DRB1* 12	3	(4)	2,5	(1)	2,5	(2)			
DR B1*13	15	(18)	10	(4)	10	(8)			
DR B1*14	5	(6)	2,5	(1)	2,5	(2)			
DRB1* 07	12	(15)	12,5	(5)	10	(8)			
DRB1* 08	7	(9)	2,5	(1)	1,3	(1)			
DRB1* 09	0	0	5	(2)	0	0			
DRB1* 10	2	(2)	0	0	0	0			
DQB1* 02	18	(22)	17,5	(7)	23	(18)			
DQB1* 03	31	(37)	45	(18)	32	(25)			
DQB1* 04	5	(6)	0	0	0	0			
DQB1* 05	23	(28)	20	(8)	31	(24)			
DQB1* 06	22	(27)	17,5	(7)	19	(15)			

## Discussion

Class II HLA molecules, by presenting antigens to helper T cells, play a decisive role in induction of antibody production [12]. Therefore, we expected to find an association of some *HLA-DRB1 *or *-DQB1 *allele(s) with the presence of anti-sperm antibodies in our patients. To our surprise, relatively weak associations were detected, showing a protective effect of HLA-DRB1*04 and HLA-DQB1*06. As our patient sample was small (23 AsA^+ ^and 37 AsA^- ^individuals), this result should be confirmed in a study of much higher number of cryptorchid patients and controls. Further to that we have to note the lack of homogeneity between compared control and tested populations illustrated at different degree of Tanner stage assigned to sexual development (see, Table 1) for both groups (AsA^+ ^versus AsA^-^). As the results listed do not indicate classical way of antisperm antibody induction along the affinity maturation (more advanced Tanner stage sexual maturity more antibodies should be elicited) we may only point out that population under study consisted of only 1 individual at Tanner stage 2 and virtually no individuals at Tanner stage 3. Antisperm antibody induction in individuals with no sperm present has been clearly shown in proteomic approach to be induced mainly by cross-reactive testicular tissue elements [13].

We have not found any association of *HLA *class II alleles with cryptorchidism itself. So far, associations of cryptorchidism with some *HLA-DRB1 *and *HLA-DQB1 *alleles, very rare in Caucasians, were described only for a Japanese population [14], whereas no correlation with *HLA *class II polymorphism was observed in a study on Northern Italian Caucasians [15]. Interestingly, this latter study reported some associations of *HLA *class I alleles (*HLA-A11, -A23 *and *-A29*), possibly explained by their crossreactivity with receptors for LH and hCG present on fetal Leydig cells and interference with the hormone-binding site through a mechanism of 'molecular mimicry' [15].

On the other hand, we obtained an unexpected finding of a strong difference between familial (but not sporadic) cryptorchidism (and/or with history of infertility within these families) and healthy controls, showing a high risk for HLA-DRB1*11 bearers. This result may suggest that sporadic and familial cryptorchidism may have different genetic background. HLA-DRB1*11 was also, albeit weakly, associated with bilateral cryptorchidism. Again, this finding should be confirmed on a larger population sample.

In summary, predisposition to produce anti-sperm antibodies seems to be only weakly associated with *HLA *class II genes, although this question requires further study on much larger population sample. It is plausible that familial and sporadic cryptorchidism may present distinct genetic background. The same may, to lower extent, apply to bilateral and unilateral cryptorchidism.

## List of abbreviations

MHC: major histocompatibility complex; IDIBT: indirect immunobead-binding test; EDTA: ethylenediaminetetraacetic acid; PCR-SSP: polymerase chain reaction-sequence-specific primer; LH: luteinizing hormone; hCG: human choriogonadotropin.

## Competing interests

The authors declare that they have no competing interests.

## Authors' contributions

MK conceived of the study, and participated in its design and coordination and helped to draft the manuscript. AN clinical supervising, sample collection. WN-M carried out molecular HLA typing. AH carried out patient documentation and helped in sample collection. MKam. carried out the detection of antisperm antibodies. BN participated in analyzing and interpretation of the data. VC helped in study coordination. PK drafted the manuscript. All authors read and approved the final manuscript.
